# Supplementary material for: Equity and Distributional Impact on Stunting of a Nutritional Package Targeting Children Aged 6–36 Months in China: Findings from a Modeling Study
Source: Nutrients. 2020 Aug 30;12(9):2643. doi: 10.3390/nu12092643 (PMC7551246; doi:10.3390/nu12092643)
Supplement: Supplementary file 1 [file nutrients-12-02643-s001.pdf]

Supplementary materials

Figure S1. Road density (km of road per 100km^2 of land area) per Chinese province, 2015

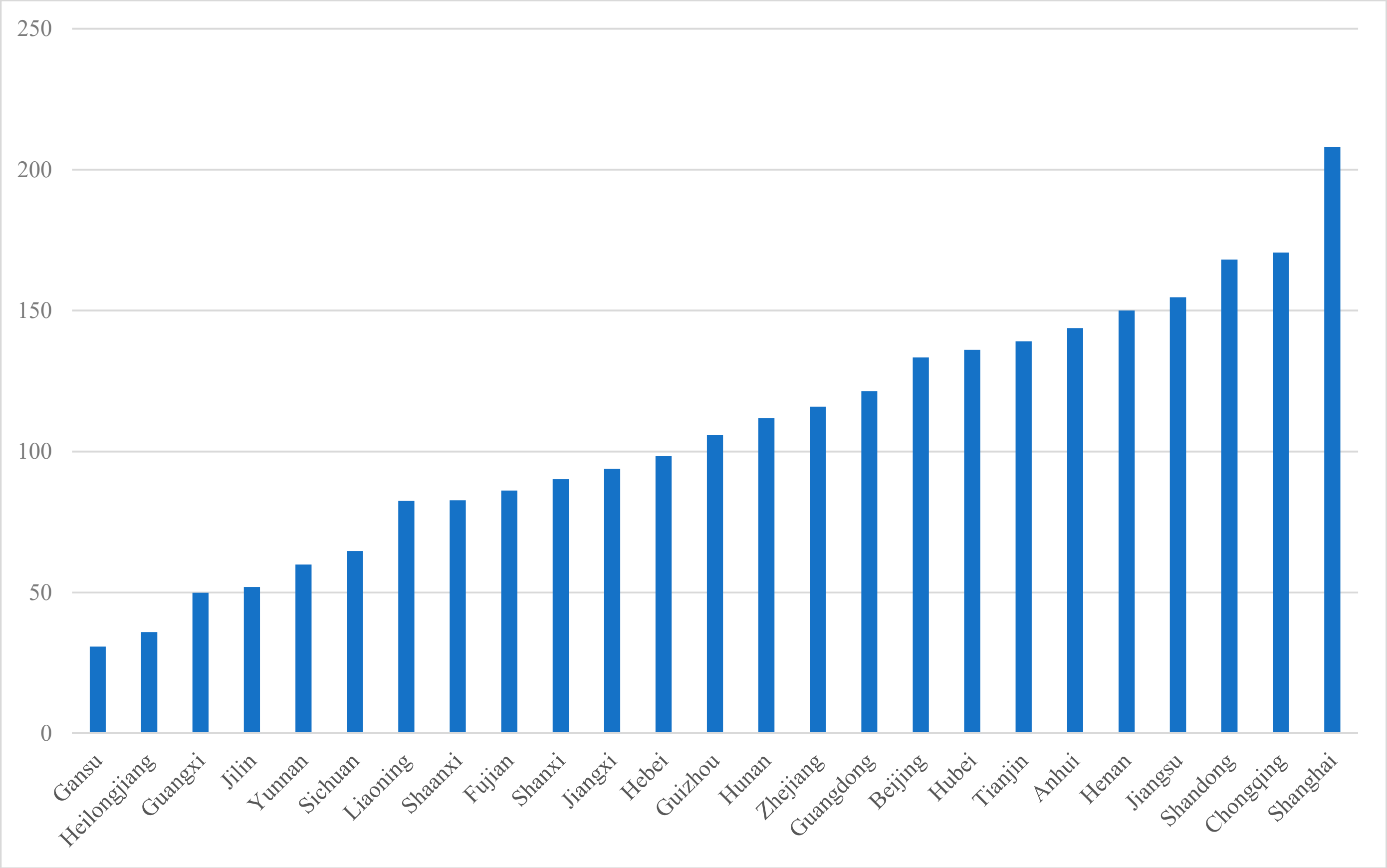

Table S1. Cost type, symbol, definition, and source

**Cost type**	**Symbol**	**Definition**	**Source**
1. Procurement and manufacturing costs	$M_{p,k}$	Includes: manufacturing, printing for educational materials, personnel training for distribution, and storage	China Development Research Foundation (1)
2. Implementation costs	$F_{p,k}$	Includes: the costs imposed on township hospitals, village health posts, and other organizations required to coordinate the delivery of YYB	China Development Research Foundation (1)
3. Advertisement costs	$A_{p,k}$	Includes: raising public awareness and training of staff for implementation	China Development Research Foundation (1)
4. Transportation costs	$T_{p,k}$	The costs to deliver YYB from manufacturing companies to eligible households	Authors' assumption
a. From manufacturing companies to township hospitals	TM_p	The costs to deliver YYB from manufacturing companies to township hospitals	Based on road density
b. From township hospitals to village health posts	TV_p	The costs to deliver YYB from township hospitals to village health posts	China Development Research Foundation (1)
c. From village health posts to eligible households	$TH_{p,k}$	The costs to deliver YYB from village health posts to eligible households (borne by caregivers)	Based on caregivers' travel time and wages
d. From township hospitals directly to eligible households	$TO_{p,k}$	The costs to deliver YYB from township hospitals to eligible households (borne by caregivers)	Based on caregivers' travel time and wages

Table S2. Estimated transportation costs per pack, per province and poverty status (cents; RMB)

Province	From manufacturing company to township hospitals (TM_p)	Caregiver transportation costs to health posts ($TH_{k,p}$)		Caregiver transportation costs to township hospitals ($TO_{k,p}$)	
		Below poverty line	Above poverty line	Below poverty line	Above poverty line
Anhui	5.5	0.8	0.3	45.8	26.1
Beijing	6.2	NA	NA	NA	NA
Chongqing	3.6	3.2	1.0	46.6	4.8
Fujian	9.6	0.5	0.4	7.0	6.2
Gansu	13.5	0.2	1.5	7.0	1.5
Guangdong	7.1	0.8	0.5	27.6	6.5
Guangxi	12.1	2.5	2.3	18.1	7.5
Guizhou	8.2	1.4	0.1	15.1	0.4
Hebei	8.7	0.7	0.7	17.3	6.7
Heilongjiang	13.1	0.5	2.2	5.5	2.9
Henan	5.0	0.1	0.3	5.1	5.0
Hubei	6.0	3.3	0.3	13.5	14.4
Hunan	7.7	1.0	2.9	21.1	17.7
Jiangsu	4.7	0.8	0.7	5.7	5.7
Jiangxi	9.0	0.9	0.8	8.1	9.2
Jilin	12.0	1.0	0.2	8.5	20.9
Liaoning	9.8	0.8	0.3	42.5	1.2
Shaanxi	9.8	0.6	0.5	20.7	12.5
Shandong	3.7	2.5	3.3	9.2	3.9
Shanghai	0.9	0.1	0.1	5.4	2.5
Shanxi	9.3	0.7	0.7	6.8	2.0
Sichuan	11.1	1.0	0.5	5.4	16.3
Tianjin	5.8	0.8	0.8	22.1	21.8
Yunnan	11.4	0.1	0.2	42.8	8.7
Zhejiang	7.5	1.8	0.7	3.1	2.7

Notes: Subsidies to village doctors to collect YYB from township hospitals to village health posts are assumed constant across provinces.

Table S3. Estimated transportation costs per pack, per province and poverty status (cents; $)

Province	From manufacturing company to township hospitals (TM_p)	Caregiver transportation costs to health posts ($TH_{k,p}$)		Caregiver transportation costs to township hospitals ($TO_{k,p}$)	
		Below poverty line	Above poverty line	Below poverty line	Above poverty line
Anhui	1.56	0.23	0.09	13.00	7.41
Beijing	1.76	NA	NA	NA	NA
Chongqing	1.02	0.91	0.28	13.22	1.36
Fujian	2.72	0.14	0.11	1.99	1.76
Gansu	3.83	0.06	0.43	1.99	0.43
Guangdong	2.01	0.23	0.14	7.83	1.84
Guangxi	3.43	0.71	0.65	5.14	2.13
Guizhou	2.33	0.40	0.03	4.28	0.11
Hebei	2.47	0.20	0.20	4.91	1.90
Heilongjiang	3.72	0.14	0.62	1.56	0.82
Henan	1.42	0.03	0.09	1.45	1.42
Hubei	1.70	0.94	0.09	3.83	4.09
Hunan	2.19	0.28	0.82	5.99	5.02
Jiangsu	1.33	0.23	0.20	1.62	1.62
Jiangxi	2.55	0.26	0.23	2.30	2.61
Jilin	3.41	0.28	0.06	2.41	5.93
Liaoning	2.78	0.23	0.09	12.06	0.34
Shaanxi	2.78	0.17	0.14	5.87	3.55
Shandong	1.05	0.71	0.94	2.61	1.11
Shanghai	0.26	0.03	0.03	1.53	0.71
Shanxi	2.64	0.20	0.20	1.93	0.57
Sichuan	3.15	0.28	0.14	1.53	4.63
Tianjin	1.65	0.23	0.23	6.27	6.19
Yunnan	3.23	0.03	0.06	12.15	2.47
Zhejiang	2.13	0.51	0.20	0.88	0.77

Notes: Subsidies to village doctors to collect YYB from township hospitals to village health posts are assumed constant across provinces.

Table S4. Estimated total transportation costs per pack by delivery type, per province and poverty status (cents; RMB)

Province	Delivery type 1 ($T_{p,k}$= TM_p+ TV_p+ $TH_{p,k}$)		Delivery type 2 ($T_{p,k}$= TM_p+ $TO_{p,k}$)	
	Below poverty line	Above poverty line	Below poverty line	Above poverty line
Anhui	7	7	51	32
Beijing	NA	NA	NA	NA
Chongqing	8	6	50	8
Fujian	11	11	17	16
Gansu	15	16	21	15
Guangdong	9	9	35	14
Guangxi	16	15	30	20
Guizhou	11	9	23	9
Hebei	10	10	26	15
Heilongjiang	15	16	19	16
Henan	6	6	10	10
Hubei	10	7	20	20
Hunan	10	12	29	25
Jiangsu	6	6	10	10
Jiangxi	11	11	17	18
Jilin	14	13	21	33
Liaoning	12	11	52	11
Shaanxi	11	11	31	22
Shandong	7	8	13	8
Shanghai	2	2	6	3
Shanxi	11	11	16	11
Sichuan	13	13	17	27
Tianjin	8	8	28	28
Yunnan	12	13	54	20
Zhejiang	10	9	11	10

Notes:
Delivery type 1: manufacturing companies deliver YYB to township hospitals; then village doctors come to township hospitals to collect YYB monthly and caregivers get YYB from village health posts.
Delivery type 2: manufacturing companies deliver YYB to township hospitals; then caregivers come to township hospitals monthly to obtain YYB.

Table S5. Estimated total transportation costs per pack by delivery type, per province and poverty status (cents; $)

Province	Delivery type 1 ($T_{p,k}$= TM_p+ TV_p+ $TH_{p,k}$)		Delivery type 2 ($T_{p,k}$= TM_p+ $TO_{p,k}$)	
	Below poverty line	Above poverty line	Below poverty line	Above poverty line
Anhui	1.99	1.99	14.47	9.08
Beijing	NA	NA	NA	NA
Chongqing	2.27	1.70	14.19	2.27
Fujian	3.12	3.12	4.82	4.54
Gansu	4.26	4.54	5.96	4.26
Guangdong	2.55	2.55	9.93	3.97
Guangxi	4.54	4.26	8.51	5.68
Guizhou	3.12	2.55	6.53	2.55
Hebei	2.84	2.84	7.38	4.26
Heilongjiang	4.26	4.54	5.39	4.54
Henan	1.70	1.70	2.84	2.84
Hubei	2.84	1.99	5.68	5.68
Hunan	2.84	3.41	8.23	7.09
Jiangsu	1.70	1.70	2.84	2.84
Jiangxi	3.12	3.12	4.82	5.11
Jilin	3.97	3.69	5.96	9.36
Liaoning	3.41	3.12	14.76	3.12
Shaanxi	3.12	3.12	8.80	6.24
Shandong	1.99	2.27	3.69	2.27
Shanghai	0.57	0.57	1.70	0.85
Shanxi	3.12	3.12	4.54	3.12
Sichuan	3.69	3.69	4.82	7.66
Tianjin	2.27	2.27	7.95	7.95
Yunnan	3.41	3.69	15.32	5.68
Zhejiang	2.84	2.55	3.12	2.84

Notes:
Delivery type 1: manufacturing companies deliver YYB to township hospitals; then village doctors come to township hospitals to collect YYB monthly and caregivers get YYB from village health posts.
Delivery type 2: manufacturing companies deliver YYB to township hospitals; then caregivers come to township hospitals monthly to obtain YYB.

Table S6. Estimated total costs per pack by delivery type, per province and poverty status (cents; RMB)

Province	Delivery type 1		Delivery type 2	
	Below poverty line	Above poverty line	Below poverty line	Above poverty line
Anhui	77	76	121	101
Beijing	NA	NA	NA	NA
Chongqing	77	75	120	78
Fujian	81	81	86	85
Gansu	84	86	90	85
Guangdong	78	78	104	83
Guangxi	85	85	100	89
Guizhou	80	79	93	78
Hebei	80	80	96	85
Heilongjiang	84	86	88	86
Henan	76	76	80	80
Hubei	80	77	89	90
Hunan	79	81	98	95
Jiangsu	76	76	80	80
Jiangxi	80	80	87	88
Jilin	84	83	90	103
Liaoning	81	81	122	81
Shaanxi	81	81	100	92
Shandong	77	78	83	77
Shanghai	72	72	76	73
Shanxi	81	81	86	81
Sichuan	83	82	86	97
Tianjin	77	77	98	97
Yunnan	82	82	124	90
Zhejiang	80	79	80	80

Notes:
Delivery type 1: manufacturing companies deliver YYB to township hospitals; then village doctors come to township hospitals to collect YYB monthly and caregivers get YYB from village health posts.
Delivery type 2: manufacturing companies deliver YYB to township hospitals; then caregivers come to township hospitals monthly to obtain YYB.

Table S7. Estimated total costs per pack by delivery type, per province and poverty status (cents; $)

Province	Delivery type 1		Delivery type 2	
	Below poverty line	Above poverty line	Below poverty line	Above poverty line
Anhui	21.9	21.6	34.3	28.7
Beijing	NA	NA	NA	NA
Chongqing	21.9	21.3	34.1	22.1
Fujian	23.0	23.0	24.4	24.1
Gansu	23.8	24.4	25.5	24.1
Guangdong	22.1	22.1	29.5	23.6
Guangxi	24.1	24.1	28.4	25.3
Guizhou	22.7	22.4	26.4	22.1
Hebei	22.7	22.7	27.2	24.1
Heilongjiang	23.8	24.4	25.0	24.4
Henan	21.6	21.6	22.7	22.7
Hubei	22.7	21.9	25.3	25.5
Hunan	22.4	23.0	27.8	27.0
Jiangsu	21.6	21.6	22.7	22.7
Jiangxi	22.7	22.7	24.7	25.0
Jilin	23.8	23.6	25.5	29.2
Liaoning	23.0	23.0	34.6	23.0
Shaanxi	23.0	23.0	28.4	26.1
Shandong	21.9	22.1	23.6	21.9
Shanghai	20.4	20.4	21.6	20.7
Shanxi	23.0	23.0	24.4	23.0
Sichuan	23.6	23.3	24.4	27.5
Tianjin	21.9	21.9	27.8	27.5
Yunnan	23.3	23.3	35.2	25.5
Zhejiang	22.7	22.4	22.7	22.7

Notes:
Delivery type 1: manufacturing companies deliver YYB to township hospitals; then village doctors come to township hospitals to collect YYB monthly and caregivers get YYB from village health posts.
Delivery type 2: manufacturing companies deliver YYB to township hospitals; then caregivers come to township hospitals monthly to obtain YYB.

Table S8. Estimated total costs for a 12-month YYB rollout by province and poverty status (25% and 75% coverage, ¥ million)

Province	25% coverage				75% coverage			
	Delivery type 1		Delivery type 2		Delivery type 1		Delivery type 2	
	Below poverty line	Above poverty line	Below poverty line	Above poverty line	Below poverty line	Above poverty line	Below poverty line	Above poverty line
Anhui	47.6	54.0	74.9	71.6	142.7	162.0	224.7	214.9
Beijing	NA	NA	NA	NA	NA	NA	NA	NA
Chongqing	18.9	18.3	29.2	19.0	56.6	55.0	87.7	57.1
Fujian	20.8	24.5	22.3	25.9	62.5	73.4	66.8	77.8
Gansu	32.2	13.9	34.5	13.7	96.6	41.6	103.4	41.1
Guangdong	60.6	48.2	80.7	51.4	181.9	144.6	242.0	154.1
Guangxi	67.5	42.3	79.2	44.4	202.6	126.9	237.6	133.3
Guizhou	52.9	23.4	61.4	23.2	158.8	70.2	184.2	69.7
Hebei	83.2	47.8	99.6	50.8	249.7	143.3	298.7	152.4
Heilongjiang	22.9	13.3	24.0	13.3	68.6	40.0	71.9	39.9
Henan	110.5	93.9	116.5	98.7	331.4	281.8	349.4	296.0
Hubei	28.9	45.9	32.3	53.8	86.8	137.7	96.9	161.4
Hunan	57.1	59.9	71.0	70.2	171.4	179.8	212.9	210.6
Jiangsu	16.8	56.0	17.7	59.0	50.4	167.9	53.1	176.9
Jiangxi	53.4	44.1	57.5	48.2	160.1	132.2	172.6	144.6
Jilin	14.2	14.1	15.3	17.4	42.6	42.2	46.0	52.3
Liaoning	16.9	14.5	25.4	14.5	50.6	43.4	76.1	43.4
Shaanxi	33.1	14.3	40.9	16.3	99.3	42.9	122.8	48.8
Shandong	67.6	55.7	72.7	55.5	202.8	167.2	218.1	166.5
Shanghai	0.1	3.3	0.1	3.4	0.4	9.9	0.4	10.1
Shanxi	24.9	19.3	26.5	19.4	74.8	58.0	79.6	58.3
Sichuan	105.1	27.4	109.6	32.3	315.4	82.1	328.8	97.0
Tianjin	1.5	4.6	1.9	5.8	4.6	13.8	5.8	17.4
Yunnan	69.8	22.2	105.4	24.2	209.4	66.6	316.2	72.7
Zhejiang	8.0	33.4	8.0	33.9	23.9	100.2	24.0	101.6
**Total**	**1,014.6**	**794.2**	**1,206.6**	**866.0**	**3,043.8**	**2,382.5**	**3,619.7**	**2,597.9**

Notes:
Delivery type 1: manufacturing companies deliver YYB to township hospitals; then village doctors come to township hospitals to collect YYB monthly and caregivers get YYB from village health posts.
Delivery type 2: manufacturing companies deliver YYBs to township hospitals; then caregivers come to township hospitals monthly to obtain YYB.

Table S9. Estimated total costs for a 12-month YYB rollout by province and poverty status (25% and 75% coverage, $ million)

Province	25% coverage				75% coverage			
	Delivery type 1		Delivery type 2		Delivery type 1		Delivery type 2	
	Below poverty line	Above poverty line	Below poverty line	Above poverty line	Below poverty line	Above poverty line	Below poverty line	Above poverty line
Anhui	13.5	15.3	21.3	20.3	40.5	46.0	63.8	61.0
Beijing	NA	NA	NA	NA	NA	NA	NA	NA
Chongqing	5.4	5.2	8.3	5.4	16.1	15.6	24.9	16.2
Fujian	5.9	7.0	6.3	7.3	17.7	20.8	19.0	22.1
Gansu	9.1	3.9	9.8	3.9	27.4	11.8	29.3	11.7
Guangdong	17.2	13.7	22.9	14.6	51.6	41.0	68.7	43.7
Guangxi	19.2	12.0	22.5	12.6	57.5	36.0	67.4	37.8
Guizhou	15.0	6.6	17.4	6.6	45.1	19.9	52.3	19.8
Hebei	23.6	13.6	28.3	14.4	70.9	40.7	84.8	43.2
Heilongjiang	6.5	3.8	6.8	3.8	19.5	11.4	20.4	11.3
Henan	31.4	26.6	33.1	28.0	94.0	80.0	99.1	84.0
Hubei	8.2	13.0	9.2	15.3	24.6	39.1	27.5	45.8
Hunan	16.2	17.0	20.1	19.9	48.6	51.0	60.4	59.8
Jiangsu	4.8	15.9	5.0	16.7	14.3	47.6	15.1	50.2
Jiangxi	15.2	12.5	16.3	13.7	45.4	37.5	49.0	41.0
Jilin	4.0	4.0	4.3	4.9	12.1	12.0	13.1	14.8
Liaoning	4.8	4.1	7.2	4.1	14.4	12.3	21.6	12.3
Shaanxi	9.4	4.1	11.6	4.6	28.2	12.2	34.8	13.8
Shandong	19.2	15.8	20.6	15.7	57.5	47.4	61.9	47.2
Shanghai	0.0	0.9	0.0	1.0	0.1	2.8	0.1	2.9
Shanxi	7.1	5.5	7.5	5.5	21.2	16.5	22.6	16.5
Sichuan	29.8	7.8	31.1	9.2	89.5	23.3	93.3	27.5
Tianjin	0.4	1.3	0.5	1.6	1.3	3.9	1.6	4.9
Yunnan	19.8	6.3	29.9	6.9	59.4	18.9	89.7	20.6
Zhejiang	2.3	9.5	2.3	9.6	6.8	28.4	6.8	28.8
**Total**	**287.9**	**225.4**	**342.4**	**245.7**	**863.7**	**676.1**	**1027.2**	**737.2**

Notes:
Delivery type 1: manufacturing companies deliver YYB to township hospitals; then village doctors come to township hospitals to collect YYB monthly and caregivers get YYB from village health posts.
Delivery type 2: manufacturing companies deliver YYBs to township hospitals; then caregivers come to township hospitals monthly to obtain YYB.

Table S10. Stunting prevalence by province and poverty status

Province	Without the rollout of YYB intervention		25% intervention coverage		75% intervention coverage	
	Below poverty line	Above poverty line	Below poverty line	Above poverty line	Below poverty line	Above poverty line
Anhui	7.1%	2.3%	6.5%	1.6%	3.3%	1.1%
Beijing	0.0%	0.0%	0.0%	0.0%	0.0%	0.0%
Chongqing	11.1%	0.0%	2.1%	0.0%	0.0%	0.0%
Fujian	15.4%	13.3%	13.6%	12.1%	8.9%	5.7%
Gansu	21.4%	16.2%	19.1%	15.5%	14.2%	13.2%
Guangdong	23.1%	17.6%	19.2%	14.5%	14.1%	9.0%
Guangxi	17.7%	13.8%	13.8%	12.8%	9.8%	5.9%
Guizhou	25.7%	15.8%	23.7%	13.8%	18.2%	9.0%
Hebei	17.9%	6.3%	13.7%	5.3%	7.2%	3.1%
Heilongjiang	22.9%	0.0%	20.1%	0.0%	11.3%	0.0%
Henan	14.5%	13.0%	13.8%	11.2%	7.2%	6.2%
Hubei	25.0%	18.0%	23.5%	17.2%	17.2%	12.3%
Hunan	18.2%	15.2%	17.3%	13.1%	9.2%	8.3%
Jiangsu	16.7%	5.0%	14.3%	4.2%	7.1%	1.0%
Jiangxi	25.6%	22.9%	20.9%	15.3%	11.8%	10.1%
Jilin	20.0%	14.3%	18.2%	13.9%	12.3%	7.2%
Liaoning	19.5%	2.3%	17.5%	1.7%	13.1%	0.0%
Shaanxi	25.0%	16.2%	22.5%	15.1%	15.4%	9.0%
Shandong	18.8%	11.5%	15.3%	11.0%	7.4%	8.1%
Shanghai	0.0%	0.0%	0.0%	0.0%	0.0%	0.0%
Shanxi	23.1%	15.8%	20.5%	14.2%	13.7%	6.7%
Sichuan	22.5%	7.7%	19.0%	2.5%	12.0%	0.0%
Tianjin	0.0%	0.0%	0.0%	0.0%	0.0%	0.0%
Yunnan	27.4%	22.0%	24.2%	20.9%	16.0%	10.6%
Zhejiang	5.9%	0.0%	3.2%	0.0%	0.0%	0.0%

Table S11. Estimated number of stunting cases by province and poverty status

Province	Before intervention		25% intervention coverage		75% intervention coverage	
	**Below poverty line**	**Above poverty line**	**Below poverty line**	**Above poverty line**	**Below poverty line**	**Above poverty line**
Anhui	48,203	17,843	44,130	12,413	22,404	8,534
Beijing	0	0	0	0	0	0
Chongqing	29,694	0	5,618	0	0	0
Fujian	43,536	44,384	38,554	40,289	25,250	18,868
Gansu	89,467	28,834	80,043	27,573	59,688	23,460
Guangdong	195,607	118,865	162,984	98,187	119,585	60,887
Guangxi	153,474	75,288	119,937	69,690	84,908	32,193
Guizhou	186,196	51,376	171,743	44,867	131,445	29,284
Hebei	204,197	40,934	156,192	34,385	82,295	20,369
Heilongjiang	68,209	0	59,899	0	33,747	0
Henan	232,063	176,300	220,502	151,584	114,675	84,347
Hubei	99,301	117,898	93,343	112,658	68,398	80,564
Hunan	143,720	122,676	136,447	106,248	72,346	67,451
Jiangsu	40,487	40,399	34,766	34,016	17,237	8,080
Jiangxi	186,086	137,474	152,252	91,936	85,982	60,807
Jilin	37,278	26,654	33,886	25,908	22,899	13,420
Liaoning	44,489	4,468	39,901	3,244	29,808	0
Shaanxi	112,066	31,405	100,860	29,331	68,994	17,447
Shandong	181,091	90,921	147,287	86,678	71,229	63,827
Shanghai	0	0	0	0	0	0
Shanxi	78,306	41,566	69,596	37,270	46,346	17,617
Sichuan	313,873	28,088	265,049	9,131	167,399	0
Tianjin	0	0	0	0	0	0
Yunnan	255,616	65,168	225,390	61,851	149,265	31,482
Zhejiang	6,456	0	3,502	0	0	0
**Total**	**2,749,415**	**1,260,542**	**2,361,878**	**1,077,258**	**1,473,900**	**638,635**

Table S12. Cost-effectiveness of YYB program, by province and poverty status (Cost [$] per stunting case averted)

Province	25% coverage				75% coverage			
	Delivery type 1		Delivery type 2		Delivery type 1		Delivery type 2	
	Below poverty line	Above poverty line	Below poverty line	Above poverty line	Below poverty line	Above poverty line	Below poverty line	Above poverty line
Anhui	3,316	2,822	5,217	3,741	1,570	4,938	2,472	6,550
Beijing	NA	NA	NA	NA	NA	NA	NA	NA
Chongqing	223	NA	344	NA	541	NA	838	NA
Fujian	1,185	1,698	1,270	1,795	970	816	1,037	865
Gansu	970	3,128	1,039	3,083	921	2,197	985	2,170
Guangdong	527	661	702	705	679	708	903	754
Guangxi	571	2,144	670	2,251	839	836	983	878
Guizhou	1,039	1,020	1,205	1,011	823	902	955	895
Hebei	492	2,071	589	2,201	581	1,977	695	2,103
Heilongjiang	782	NA	820	NA	565	NA	592	NA
Henan	2,712	1,078	2,860	1,133	801	870	844	913
Hubei	1,377	2,486	1,538	2,913	797	1,047	890	1,227
Hunan	2,228	1,035	2,770	1,213	681	924	846	1,082
Jiangsu	833	2,490	878	2,623	615	1,474	648	1,553
Jiangxi	448	275	482	300	454	489	489	535
Jilin	1,188	5,364	1,280	6,619	841	905	908	1,121
Liaoning	1,045	3,362	1,571	3,362	978	2,757	1,471	2,757
Shaanxi	838	1,957	1,036	2,230	654	872	809	992
Shandong	568	3,725	610	3,712	524	1,751	563	1,744
Shanghai	NA	NA	NA	NA	NA	NA	NA	NA
Shanxi	811	1,275	863	1,281	664	687	707	691
Sichuan	611	410	637	484	611	829	637	980
Tianjin	NA	NA	NA	NA	NA	NA	NA	NA
Yunnan	655	1,899	990	2,070	559	561	844	612
Zhejiang	768	NA	768	NA	1,051	NA	1,055	NA

Notes:

Delivery type 1: manufacturing companies deliver YYB to township hospitals; then village doctors come to township hospitals to collect YYB monthly and caregivers get YYB from village health posts.

Delivery type 2: manufacturing companies deliver YYBs to township hospitals; then caregivers come to township hospitals monthly to obtain YYB.

Table S13. Cost-effectiveness of YYB program, by province and poverty status, when the effect size of YYB is halved (Cost [$] per stunting case averted)

Province	25% coverage				75% coverage			
	Delivery type 1		Delivery type 2		Delivery type 1		Delivery type 2	
	Below poverty line	Above poverty line	Below poverty line	Above poverty line	Below poverty line	Above poverty line	Below poverty line	Above poverty line
Anhui	5,227	10,945	8,228	14,523	3,136	6,896	4,937	9,147
Beijing	NA	NA	NA	NA	NA	NA	NA	NA
Chongqing	452	NA	700	NA	1,131	NA	1,753	NA
Fujian	1,890	4,574	2,022	4,854	1,797	1,719	1,922	1,824
Gansu	1,652	12,562	1,768	12,460	1,960	4,280	2,098	4,235
Guangdong	1,240	1,444	1,650	1,538	1,288	1,358	1,713	1,446
Guangxi	1,075	3,160	1,260	3,321	1,754	1,729	2,057	1,817
Guizhou	1,660	2,721	1,926	2,701	1,607	1,975	1,864	1,960
Hebei	1,147	3,230	1,373	3,436	1,166	3,420	1,396	3,639
Heilongjiang	1,464	NA	1,535	NA	1,124	NA	1,179	NA
Henan	5,512	2,745	5,811	2,882	1,534	1,732	1,618	1,818
Hubei	3,156	4,502	3,523	5,277	1,600	2,101	1,786	2,463
Hunan	3,515	2,469	4,367	2,892	1,428	1,803	1,774	2,112
Jiangsu	1,356	2,857	1,427	3,011	1,307	2,879	1,376	3,034
Jiangxi	817	566	881	619	909	978	980	1,070
Jilin	2,575	10,727	2,778	13,273	1,731	1,679	1,868	2,081
Liaoning	4,102	7,662	6,166	7,662	2,412	5,334	3,626	5,334
Shaanxi	1,750	4,062	2,166	4,621	1,268	1,775	1,569	2,020
Shandong	1,120	9,459	1,205	9,419	1,053	4,020	1,133	4,005
Shanghai	NA	NA	NA	NA	NA	NA	NA	NA
Shanxi	1,465	3,666	1,560	3,682	1,308	1,333	1,392	1,340
Sichuan	1,268	872	1,322	1,031	1,290	1,709	1,344	2,020
Tianjin	NA	NA	NA	NA	NA	NA	NA	NA
Yunnan	1,198	2,577	1,808	2,817	1,091	1,175	1,647	1,284
Zhejiang	1,577	NA	1,585	NA	2,297	NA	2,308	NA

Notes:

Delivery type 1: manufacturing companies deliver YYB to township hospitals; then village doctors come to township hospitals to collect YYB monthly and caregivers get YYB from village health posts.

Delivery type 2: manufacturing companies deliver YYB to township hospitals; then caregivers come to township hospitals monthly to obtain YYB.

Table S14. Cost-effectiveness of YYB program, by province and poverty status, when the effect size of YYB is reduced to 0.96cm (Cost [$] per stunting case averted)

	25% coverage				75% coverage			
	Delivery type 1		Delivery type 2		Delivery type 1		Delivery type 2	
Province	Below poverty line	Above poverty line	Below poverty line	Above poverty line	Below poverty line	Above poverty line	Below poverty line	Above poverty line
Anhui	5,924	15,797	9,622	17,336	3,440	8,102	6,089	10,033
Beijing	NA	NA	NA	NA	NA	NA	NA	NA
Chongqing	549	NA	739	NA	1,362	NA	1,880	NA
Fujian	2,341	5,584	2,270	5,079	2,026	1,934	1,998	2,202
Gansu	1,756	13,104	2,115	16,065	2,610	5,000	2,655	5,559
Guangdong	1,530	1,533	2,274	1,681	1,491	1,598	1,965	1,593
Guangxi	1,228	3,589	1,537	3,531	1,868	1,982	2,424	2,171
Guizhou	1,964	3,098	2,178	3,288	1,919	2,213	2,395	2,382
Hebei	1,388	3,869	1,535	3,825	1,549	3,911	1,579	4,080
Heilongjiang	1,890	NA	1,952	NA	1,179	NA	1,297	NA
Henan	6,963	3,494	7,730	3,027	1,614	2,089	1,854	1,942
Hubei	3,248	4,760	3,737	5,336	1,918	2,275	2,330	3,014
Hunan	3,664	2,814	5,019	3,686	1,932	2,287	2,196	2,483
Jiangsu	1,535	3,008	1,591	3,185	1,669	2,951	1,696	3,761
Jiangxi	879	692	1,018	740	1,120	1,031	1,257	1,227
Jilin	3,102	13,249	2,916	17,026	1,931	2,010	1,914	2,367
Liaoning	6,055	8,296	7,555	9,704	3,058	6,530	4,106	6,861
Shaanxi	2,323	5,182	2,211	5,022	1,493	2,104	1,963	2,612
Shandong	1,306	11,760	1,541	12,161	1,329	5,410	1,488	5,460
Shanghai	NA	NA	NA	NA	NA	NA	NA	NA
Shanxi	1,688	5,211	1,985	4,287	1,614	1,570	1,657	1,606
Sichuan	1,645	1,014	1,641	1,124	1,454	1,962	1,680	2,493
Tianjin	NA	NA	NA	NA	NA	NA	NA	NA
Yunnan	1,548	2,923	2,100	3,084	1,167	1,466	1,958	1,548
Zhejiang	2,110	NA	1,809	NA	2,542	NA	2,715	NA

Notes:
Delivery type 1: manufacturing companies deliver YYB to township hospitals; then village doctors come to township hospitals to collect YYB monthly and caregivers get YYB from village health posts.
Delivery type 2: manufacturing companies deliver YYB to township hospitals; then caregivers come to township hospitals monthly to obtain YYB.

Table S15. Cost-effectiveness of YYB program, by province and poverty status, when the transportation costs (both for delivery type 1 and delivery type 2) are halved or doubled (Cost [$] per stunting case averted)

Province	25% coverage				75% coverage			
	Delivery type 1		Delivery type 2		Delivery type 1		Delivery type 2	
	Below poverty line	Above poverty line	Below poverty line	Above poverty line	Below poverty line	Above poverty line	Below poverty line	Above poverty line
	The cost per stunting case averted assuming the transportation costs of YYB were halved							
Anhui	3,159	2,699	4,111	3,159	1,496	4,722	1,947	5,528
Beijing	NA	NA	NA	NA	NA	NA	NA	NA
Chongqing	211	NA	272	NA	514	NA	663	NA
Fujian	1,105	1,580	1,146	1,631	903	761	937	785
Gansu	885	2,828	919	2,812	841	1,990	873	1,979
Guangdong	498	626	585	647	641	669	753	692
Guangxi	519	1,951	569	2,005	762	760	834	781
Guizhou	971	960	1,054	957	769	849	835	845
Hebei	460	1,936	509	2,002	544	1,849	601	1,912
Heilongjiang	713	NA	732	NA	516	NA	530	NA
Henan	2,604	1,035	2,678	1,062	769	834	791	856
Hubei	1,289	2,369	1,370	2,583	746	998	792	1,088
Hunan	2,094	962	2,364	1,051	640	858	723	937
Jiangsu	799	2,385	821	2,452	590	1,413	606	1,453
Jiangxi	417	256	435	269	423	457	441	480
Jilin	1,089	4,930	1,136	5,570	771	833	804	942
Liaoning	970	3,123	1,232	3,123	909	2,567	1,155	2,567
Shaanxi	780	1,820	879	1,954	608	811	686	871
Shandong	541	3,537	563	3,530	499	1,662	519	1,658
Shanghai	NA	NA	NA	NA	NA	NA	NA	NA
Shanxi	757	1,191	783	1,194	619	641	640	642
Sichuan	563	379	576	416	563	766	576	841
Tianjin	NA	NA	NA	NA	NA	NA	NA	NA
Yunnan	606	1,754	773	1,841	516	518	659	544
Zhejiang	717	NA	718	NA	984	NA	986	NA

Notes:
Delivery type 1: manufacturing companies deliver YYB to township hospitals; then village doctors come to township hospitals to collect YYB monthly and caregivers get YYB from village health posts.
Delivery type 2: manufacturing companies deliver YYB to township hospitals; then caregivers come to township hospitals monthly to obtain YYB.

Table S15 (continued). Cost-effectiveness of YYB program, by province and poverty status, when the transportation costs (both for delivery type 1 and delivery type 2) are halved or doubled (Cost [$] per stunting case averted)

Province	25% coverage				75% coverage			
	Delivery type 1		Delivery type 2		Delivery type 1		Delivery type 2	
	Below poverty line	Above poverty line	Below poverty line	Above poverty line	Below poverty line	Above poverty line	Below poverty line	Above poverty line
	The cost per stunting case averted assuming the transportation costs of YYB were doubled							
Anhui	3,625	3,070	7,432	4,912	1,717	5,373	3,520	8,597
Beijing	NA	NA	NA	NA	NA	NA	NA	NA
Chongqing	245	NA	489	NA	595	NA	1,190	NA
Fujian	1,348	1,924	1,512	2,131	1,102	927	1,236	1,026
Gansu	1,138	3,698	1,274	3,633	1,080	2,602	1,209	2,556
Guangdong	587	734	935	820	755	785	1,204	877
Guangxi	675	2,531	873	2,748	991	986	1,281	1,071
Guizhou	1,176	1,139	1,508	1,123	931	1,007	1,194	993
Hebei	555	2,336	749	2,600	656	2,232	885	2,484
Heilongjiang	915	NA	991	NA	662	NA	717	NA
Henan	2,927	1,167	3,221	1,275	865	941	952	1,028
Hubei	1,554	2,719	1,875	3,573	899	1,145	1,084	1,505
Hunan	2,499	1,182	3,580	1,537	764	1,055	1,094	1,371
Jiangsu	904	2,694	992	2,963	667	1,596	732	1,756
Jiangxi	508	311	578	362	515	554	586	646
Jilin	1,386	6,201	1,573	8,765	981	1,048	1,114	1,482
Liaoning	1,192	3,809	2,242	3,809	1,117	3,131	2,102	3,131
Shaanxi	955	2,226	1,353	2,764	745	993	1,056	1,232
Shandong	620	4,107	706	4,078	572	1,929	652	1,916
Shanghai	NA	NA	NA	NA	NA	NA	NA	NA
Shanxi	922	1,450	1,027	1,463	754	780	839	787
Sichuan	707	472	759	621	707	956	759	1,257
Tianjin	NA	NA	NA	NA	NA	NA	NA	NA
Yunnan	755	2,187	1,423	2,539	643	646	1,213	750
Zhejiang	863	NA	871	NA	1,185	NA	1,196	NA

References

1. China Development Research Foundation. One yuan Yingyangbao program [Internet]. Available from: http://www.ccgp-gansu.gov.cn/upload/article/20180307172835.pdf (Accessed on August 27th, 2020)
